# Mortality Disparities Among Arrestees by Race, Sentencing Disposition, and Place

**DOI:** 10.1001/jamahealthforum.2024.1794

**Published:** 2024-07-12

**Authors:** George Zuo, Beau Kilmer, Nancy Nicosia

**Affiliations:** 1 RAND Corporation, Pittsburgh, Pennsylvania

## Abstract

**Importance:**

Understanding mortality disparities among justice system–involved populations is crucial for public health and policy, especially for marginalized racial groups such as American Indian/Alaska Native persons.

**Objective:**

To examine racial disparities in mortality within the broader justice system–involved population in South Dakota, focusing on different sentencing dispositions and the role of place.

**Design, Setting, and Participants:**

This observational study used administrative criminal records linked to mortality data from January 2000 to December 2016. The statewide data linked data from South Dakota Attorney General’s Office and South Dakota Department of Health. Individuals aged 18 years and older with arrests were analyzed in this population-based sample. Data were analyzed from August 1, 2022, to July 30, 2023.

**Exposure:**

Sentencing dispositions were categorized as arrest only, fine, probation, jail, and prison.

**Main Outcomes and Measures:**

The main outcomes were mortality rates (both all-cause and cause-specific) calculated using Poisson regression models, adjusted for demographic and county variables.

**Results:**

Of 182 472 individuals with 422 987 arrests, the study sample included 29 690 American Indian/Alaska Native arrestees (17 900 [60%] male; mean [SD] age, 29.4 [11.0] years) and 142 248 White arrestees (103 471 [73%] male; mean [SD] age, 32.6 [12.9] years). American Indian/Alaska Native persons accounted for 16% of arrestees and 26% of arrests, but only 9% of the population in South Dakota. Across dispositions, mortality risk was greater for White individuals sentenced to probation, jail, and prison relative to White individuals who were arrested only. In terms of racial disparities, all-cause mortality risk was 2.37 (95% CI, 1.95-2.88) times higher for American Indian/Alaska Native than White arrestees in the arrest-only disposition. Disparities persisted across all dispositions but narrowed substantially for probation and prison. Results were similar for cause-specific mortality risk, except for cancer risk. In urban areas, mortality risk was 2.70 (95% CI, 1.29-2.44) times greater for American Indian/Alaska Native individuals relative to White individuals among those with arrest-only dispositions.

**Conclusions and Relevance:**

In this population-based observational study, mortality risk and associated racial disparities among justice system–involved individuals differed substantially across dispositions and places, underscoring the need for public health interventions tailored to these factors. Further research is needed to understand the mechanisms through which sentencing and place shape these disparities.

## Key Points

**Question:**

How does mortality risk among justice system–involved individuals vary by race, sentencing disposition (ie, prison, jail, probation, fines, arrest only), and place?

**Findings:**

In this observational study analyzing 182 472 unique arrestees in South Dakota from 2000 to 2016, large racial disparities in mortality risk are documented between American Indian or Alaska Native individuals across all sentencing dispositions. Racial disparities were significantly smaller for those with probation and prison sentences, particularly in urban areas.

**Meaning:**

Justice-involved individuals experience noteworthy racial disparities in mortality that vary by sentence and place, underscoring the need for public health interventions tailored to these factors.

## Teaser

This population-based observational study assesses disparities in mortality risk between American Indian/Alaska Native and White arrestees in South Dakota from 2000 to 2016.

## Introduction

Despite the important implications of health disparities among the justice system–involved population in the US, substantial gaps in our understanding remain.^1,2^ Conceptually, the link between criminal justice involvement and health disparities is complex. While criminal justice involvement could improve health through increased access to care and removal from harmful environments, it could also disrupt continuity of care, increase financial strain, and expose individuals to low-quality services and higher risks for disease and violence within jails and prisons.^1,3^ Empirical research has also long established that racial minority individuals exhibit higher mortality risk, experience disproportionate involvement in the justice system, and face greater disadvantages in the community.^4,5,6,7^ However, efforts to examine the intersection of these issues have been limited.

First, most studies of mortality among justice system–involved individuals in the US focus on current or recently released prisoners, even though the prison population represents only a small (less than 5%) and unrepresentative share of the justice system–involved population.^8,9,10,11,12,13,14,15,16,17^ As a result, the empirical literature has little to say about the larger and broader justice system–involved population, such as those sentenced to jail, probation, financial sanctions, and those arrested but not convicted.^18,19,20,21^ Second, despite the emergence of place as a key determinant of health, little is known about how places shape mortality risk among justice system–involved individuals.^22^ The small literature exploring the issue of place with respect to health outcomes among justice system–involved individuals also focuses on former prisoners.^23,24^ Finally, severely disadvantaged populations, such as the justice system–involved American Indian/Alaska Native population, are often overlooked. American Indian/Alaska Native individuals are one-third more likely to be arrested than White individuals, twice as likely to be in jail, and more than 4 times as likely to be in prison.^25,26,27^ In the general population, American Indian/Alaska Native mortality rates are also 40% higher than White mortality rates and 17% higher than Black mortality rates.^28^

Our empirical analysis quantifies how the net effect on mortality across the various mechanisms differs between American Indian/Alaska Native and White justice system–involved individuals. This population-based observational study provides early evidence to demonstrate racial disparities in mortality within the broader justice system–involved population and how these disparities differ across sentencing dispositions and place, particularly in the American Indian/Alaska Native population.

## Methods

### Study Design and Data Collection

This analysis used criminal records from the South Dakota Attorney General’s Office, which were linked to mortality records from the South Dakota Department of Health. The criminal records data contain 422 987 arrests from 2000 to 2016 among 182 472 unique individuals ages 18 and older. Each arrest record includes the individual’s race, sex, sentencing outcome (hereafter, disposition), arrest and disposition dates, offenses, and arrest county. Data on race and sex are entered by the arresting officer. From these records, we are also able to construct the individual’s entire South Dakota criminal history. Linked mortality records capture the date, cause of death, and limited demographic information. Our procedure yielded 5618 matches based on combinations of social security number, first and last name, and date of birth (eAppendix 1 in Supplement 1).

To compare mortality rates between justice system–involved individuals and South Dakota’s general population, we analyzed restricted-use mortality microdata from the Centers for Disease Control and Prevention’s National Vital Statistics System from 2000 to 2016. These data provide key characteristics including age, sex, race, and residence county.

This cohort study meets the Strengthening the Reporting of Observational Studies in Epidemiology (STROBE) reporting guideline.^29^ The study was approved by the RAND Institutional Review Board. Informed consent was waived because this was a retrospective study using secondary data.

### Statistical Analysis

We first calculated mortality rates by dividing the number of deaths by the number of person-years at risk. Time-at-risk begins with the individual’s first (ie, index) arrest during the analysis period and ends with death or the conclusion of the study period (December 31, 2016). We categorize each individual’s index arrest into 1 of 5 distinct sentencing dispositions (in order of increasing severity): (1) arrest only (ie, no conviction/sentence), (2) fine/restitution (hereafter fine, but no probation or incarceration), (3) probation (no incarceration), (4) jail (no prison), and (5) prison.

We also conducted sensitivity analyses using an alternative approach, in which an individual’s index arrest (and their assigned disposition) is based on their most recent arrest instead of their first arrest only, thereby allowing assigned dispositions to change over time (eAppendix 4 in Supplement 1). In these analyses, time-at-risk for a given disposition begins with that arrest and ends with a subsequent arrest, death, or the end of the sample period.

Similar to past studies linking incarceration to mortality, we compared aggregate mortality rates between justice system–involved individuals and South Dakota residents from 2000 to 2016.^8,9,11,12,13,15,30^ We use indirect standardization, or binning by age, sex, and county type (ie, urban, rural part–Indian Country, rural non–Indian Country), to compare observed vs expected mortality rates in the arrested population across dispositions. To calculate the number of expected deaths for each age/sex/county type cell, we multiplied the cell-specific mortality rate from the broader South Dakota population (subtracting out deaths from justice system–involved individuals to avoid double counting) by the total number of person-years for the corresponding group of individuals in the arrestee population. We then compared expected deaths against observed deaths.

Next, we estimated Poisson regression models to quantify mortality risk and racial disparities across disposition categories using time at risk as the exposure variable.^31^ All models controlled for categorical age, sex, offense type (drug, violent, property, driving under the influence), number of prior arrests, arrest year, and county type. The primary variables of interest are a dichotomous indicator for American Indian/Alaska Native race, a categorical variable encompassing the 5 sentencing dispositions, and their interactions. Together, these coefficients fully characterize regression-adjusted mortality by race and sentencing disposition.

Next, to explore potential mechanisms, we reestimated models for cause-specific mortality for 4 broad causes observed in our data: external causes (24.7%), cancer (19.2%), cardiovascular/respiratory disease (23.7%), and other causes (32.4%) (eAppendix 2 in Supplement 1 for *International Statistical Classification of Diseases and Related Health Problems, Tenth Revision [ICD-10]* codes).

Finally, to assess the role of place, we reestimated all-cause mortality models separately by county type (eAppendix 3 in Supplement 1). County type provides a summary measure of structural and economic characteristics that have been used in similar criminal justice studies.^32^ Counties in the South Dakota sample were classified as urban (8 counties), rural part–Indian Country (17 counties), and rural non–Indian Country (35 counties) (eFigure 1 in Supplement 1). Analyses were conducted using Stata 18/MP (StataCorp LLC).

## Results

Of 182 472 individuals with 422 987 arrests, the study sample included 29 690 American Indian/Alaska Native arrestees (17 900 [60%] male; mean [SD] age, 29.4 [11.0] years) and 142 248 White arrestees (103 471 [73%] male; mean [SD] age, 32.6 [12.9] years). American Indian/Alaska Native persons are overrepresented in the arrest data, accounting for 17% of unique arrestees and 26% of arrests but only 9% of the South Dakota population.^33^ Unadjusted deaths per person-year were 47% higher among American Indian/Alaska Native relative to White arrestees from 2000 to 2016 (Table 1). More than 80% of arrests of White individuals in the sample did not lead to an incarceration sentence: 23% were not convicted; 6% were fined; 52% were sentenced to probation; 16% were sentenced to jail; and 3% were sentenced to prison. The only significant racial differences in disposition are the higher rate of jail and a lower rate of probation sentences for American Indian/Alaska Native arrestees relative to White arrestees. Demographically, American Indian/Alaska Native arrestees were more likely to be young and female. The urban/rural split is comparable for both racial groups, although rural American Indian/Alaska Native individuals were less likely to be arrested in non–Indian Country than rural White individuals.

**Table 1.** Characteristics of American Indian/Alaska Native and White Arrestees in South Dakota, 2000-2016

Characteristic	No. (%) (N = 171 938)		
	White	American Indian/Alaska Native	*P* value
Total arrestees	142 248 (83)	29 690 (17)	NA
Mortality			
Arrestees with observed death	4195 (3)	1307 (4)	<.001
Deaths per 100 000 person-years, No.	317	467	<.001
Age group, y			
18-24	57 030 (40)	14 175 (48)	<.001
25-34	31 948 (22)	7514 (25)	<.001
35-44	25 985 (18)	4802 (16)	<.001
45-54	18 176 (13)	2295 (08)	<.001
≥55	9109 (6)	904 (03)	<.001
Sex			
Female	38 777 (27)	11 790 (40)	<.001
Male	103 471 (73)	17 900 (60)	<.001
Sentencing outcome			
Arrest only	32 603 (23)	7066 (24)	.47
Fine/restitution	8623 (6)	1571 (05)	.64
Probation	74 590 (52)	12 511 (42)	<.001
Jail	22 162 (16)	7518 (25)	<.001
Prison	4270 (3)	1024 (03)	.14
Arrest characteristics			
Driving under the influence	65 466 (46)	9851 (33)	<.001
Violent	19 175 (13)	5304 (18)	<.001
Property	21 565 (15)	6393 (22)	<.001
Drug	27 895 (20)	5047 (17)	.01
Has a pre-2000 arrest	28 614 (20)	8004 (27)	<.001
County of arrest			
Urban	70 190 (49)	15 109 (51)	.91
Rural, non–Indian Country	54 779 (39)	5658 (19)	.04
Rural, Indian Country	17 279 (12)	8923 (30)	.11

Abbreviation: NA, not applicable.

**Figure 1.**
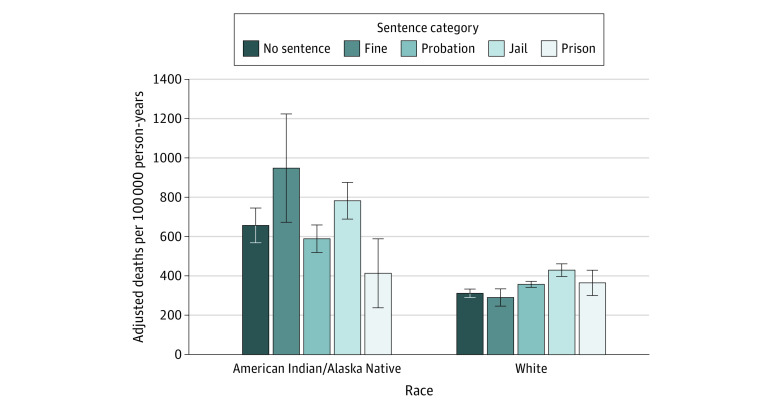
Adjusted Mortality Rates by Race and Sentence Category This figure presents age-adjusted and sex-adjusted mortality rates (reference group: White) across 5 different sentencing dispositions, referencing American Indian/Alaska Native and White arrestees’ first arrest between 2000 and 2016. The error bars represent 95% CIs.

Figure 1 compares age-adjusted and sex-adjusted mortality rates across dispositions for American Indian/Alaska Native and White arrestees. For both groups, mortality rates are higher among those sentenced to jail compared with probation. For American Indian/Alaska Native arrestees only, mortality rates are lower in prison than in jail. In sensitivity analyses, which used dispositions for each individual’s most recent arrest (vs first arrest only), the patterns and magnitudes are nearly unchanged for both American Indian/Alaska Native and White arrestees (eFigure 2 in Supplement 1).

**Figure 2.**
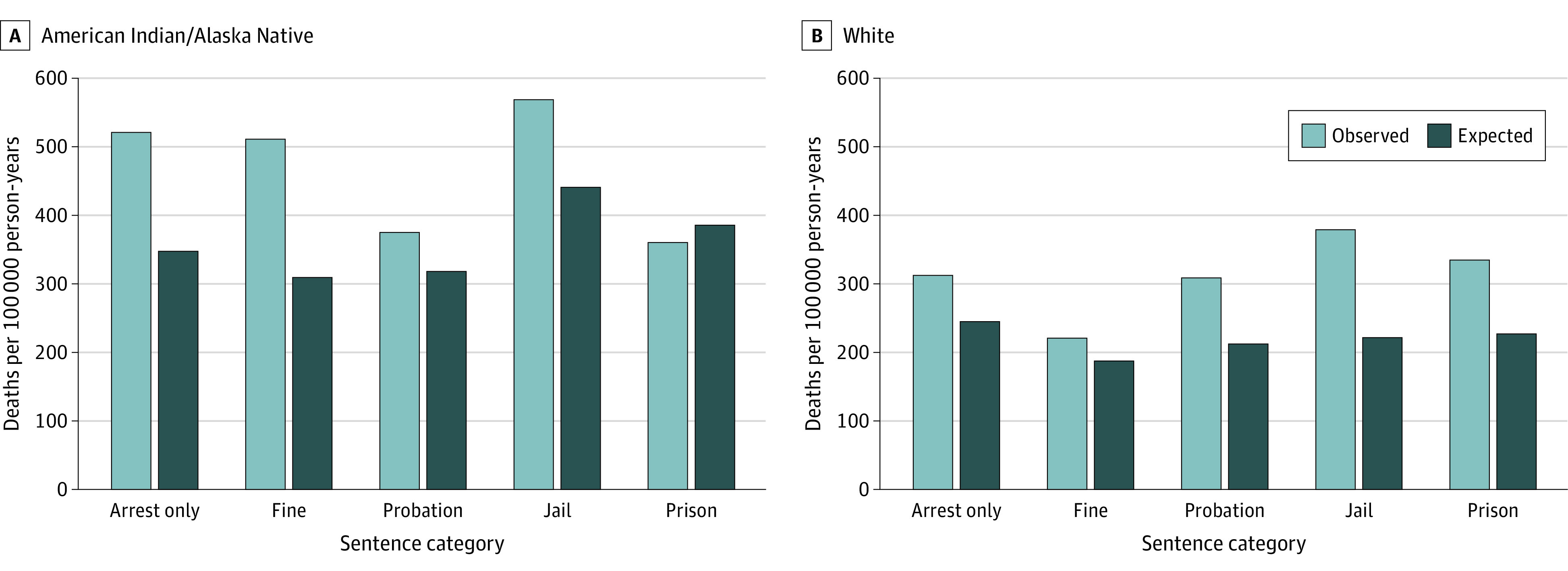
Observed vs Expected Mortality Rates by Race and Disposition This figure presents observed mortality rates by race and sentencing disposition for South Dakota’s criminal justice population between 2000 and 2016. Observed mortality rates are compared against expected mortality rates calculated based on the age, sex, and geographic composition for each subgroup and determining the expected number of deaths that would have occurred for an equivalent mix of age/sex/location in the broader non–justice system–involved South Dakota population.

Figure 2 compares observed with expected mortality rates for each disposition based on age, sex, and county type in the broader population of same-race South Dakota residents. Among White arrestees, observed mortality is significantly higher than expected mortality for arrest only (>10%), probation (>25%), jail (>47%), and prison sentences (>25%). The pattern suggests that White arrestees face greater mortality risk compared to the typical White resident of South Dakota. For American Indian/Alaska Native arrestees, observed mortality is significantly lower than expected mortality among those sentenced to probation (−22%) and prison (−36%). This pattern is robust to sensitivity analyses using dispositions from individuals’ most recent arrest (eFigure 3 in Supplement 1).

The Poisson regression model analyzes mortality risk for race, disposition, and their interactions while controlling for demographic, arrest, criminal history characteristics, and county type (Table 2). Each estimate reflects the multiplicative effect of the corresponding characteristic relative to the reference race (White) and reference disposition (arrest only).

**Table 2.** Regression-Adjusted Racial Mortality Disparities by Disposition and Cause of Death^a^

Variable/interaction	Incidence rate ratio (95% CI)				
	All deaths	Cause-specific deaths			
		External causes	Cancer	Circulatory/respiratory	Other
Arrestees, No.	171 918	171 918	171 918	171 918	171 918
Person-years, No.	1 603 821	1 603 821	1 603 821	1 603 821	1 603 821
Disposition					
How mortality risk of White individuals differs by disposition, relative to arrest only					
Fine	0.94 (0.79-1.11)	0.78 (0.59-1.02)	0.80 (0.51-1.26)	1.09 (0.85-1.41)	1.09 (0.68-1.76)
Probation	1.18 (1.05-1.31)^c^	1.09 (0.94-1.27)	1.20 (1.04-1.39)^b^	1.23 (0.99-1.51)	1.18 (1.00-1.38)^b^
Jail	1.45 (1.26-1.67)^c^	1.23 (0.98-1.54)	1.36 (1.10-1.68)^c^	1.62 (1.23-2.13)^c^	1.58 (1.21-2.06)^c^
Prison	1.19 (1.03-1.39)^b^	1.09 (0.87-1.36)	1.29 (0.95-1.75)	1.24 (0.89-1.71)	1.17 (0.87-1.57)
Racial disparities					
Baseline racial disparities for arrest-only reference disposition					
American Indian/Alaska Native individuals (arrest only)	2.37 (1.95-2.88)^c^	2.44 (1.83-3.25)^c^	0.69 (0.39-1.19)	1.87 (1.47-2.38)^c^	4.33 (3.59-5.24)^c^
How baseline racial disparities are modified for each disposition					
Fine × American Indian/Alaska Native individuals	1.21 (0.97-1.50)	1.14 (0.78-1.68)	2.85 (1.24-6.56)^b^	1.41 (0.95-2.10)	0.90 (0.52-1.57)
Probation × American Indian/Alaska Native individuals	0.72 (0.58-0.89)^c^	0.62 (0.45-0.85)^c^	1.56 (0.79-3.08)	0.67 (0.51-0.88)^c^	0.74 (0.56-0.98)^b^
Jail × American Indian/Alaska Native individuals	0.81 (0.69-0.95)^c^	0.92 (0.70-1.22)	1.41 (0.83-2.39)	0.81 (0.60-1.10)	0.66 (0.46-0.94)^b^
Prison × American Indian/Alaska Native individuals	0.63 (0.45-0.89)^c^	0.61 (0.38-0.97)^b^	0.62 (0.22-1.80)	0.72 (0.33-1.55)	0.64 (0.35-1.19)

^a^ This table presents results from interacted Poisson regressions of mortality on race (reference group: White) and sentencing disposition (reference group: no disposition), controlling for sex, age group, offense type (drug/driving under the influence/violent/property), number of prior arrests, county type (urban/rural non–Indian Country/rural part–Indian Country), and arrest year. Standard errors were clustered at the county level.

^b^ *P* < .05.

^c^ *P* < .01.

The first set of estimates indicates how mortality risk among White arrestees varies by disposition relative to the arrest-only reference disposition: 1.18 (95% CI, 1.05-1.31) times greater mortality risk for probation sentences, 1.45 (95% CI, 1.26-1.67) times greater for jail sentences, and 1.19 (95% CI,1.03-1.39) times greater for prison sentences.

The second set of estimates speaks to racial disparities. American Indian/Alaska Native mortality rates were 2.37 (95% CI, 1.95-2.88) times greater than White mortality rates for the arrest-only disposition (reference category). The interaction terms indicate the relative size of racial disparities across each disposition compared to the arrest-only reference disposition. Racial disparities were 0.72 (95% CI, 0.58-0.89) times smaller for probation sentences, 0.81 (95% CI, 0.69-0.95) times smaller for jail sentences, and 0.63 (95% CI, 0.45-0.89) times smaller for those with prison sentences.

These components can be used to calculate racial disparities within and across dispositions (see eAppendix 5 in Supplement 1 for discussion of calculations). Of particular interest are the within-disposition racial disparities: that is, the comparison between American Indian/Alaska Native and White individuals sentenced to the same disposition. For example, the racial disparity for those sentenced to probation is simply the racial disparity for the reference group multiplied by the interaction term for probation and race (2.37 × 0.72). These calculations indicate that within-disposition racial disparities persist across all dispositions, even though the gap narrows substantially for prison and probation (eTable 3 in Supplement 1). Racial disparities can also be calculated across dispositions. For example, an American Indian/Alaska Native arrestee with a prison sentence experiences 1.78 times greater all-cause mortality risk than a White arrestee with an arrest-only disposition because of the higher risk for American Indian/Alaska Native relative to White individuals with an arrest-only disposition (2.37 [95% CI, 1.95-2.88]), the higher risk for White arrestees with a prison sentence (1.19 [95% CI, 1.03-1.39]), and the relative risk of prison for American Indian/Alaska Native arrestees (0.63 [95% CI, 0.45-0.89]).

To investigate potential mechanisms, the next columns reestimate the regressions for major causes of death (Table 2). Mortality risk among White arrestees does not differ across dispositions for external causes but is elevated for cancer, circulatory/respiratory, and other causes among those with jail sentences. It is also elevated for cancer and other causes among those with probation sentences. Regarding racial disparities, American Indian/Alaska Native individuals with arrest-only dispositions were 2.44 (95% CI, 1.83-3.25) times more likely to die of external causes, 1.87 (95% CI, 1.47-2.38) times more likely to die of circulatory/respiratory diseases, and 4.33 (95% CI, 3.59-5.24) times more likely to die of other causes. The interaction terms indicated that for external causes, racial disparities narrowed among those sentenced to probation (incidence rate ratio [IRR], 0.62 [95% CI, 0.45-0.85]) and prison (IRR, 0.61 [95% CI, 0.38-0.97]). Disparities also decreased for circulatory/respiratory mortality among those sentenced to probation (IRR, 0.67 [95% CI, 0.51-0.88]), and similar decreases are observed for other causes of death among those sentenced to probation (IRR, 0.74 [95% CI, 0.56-0.98]) and jail (IRR, 0.66 [95% CI, 0.46-0.94]). Racial disparities for cancer mortality increased among those sentenced to a fine, though the confidence intervals are wide (IRR, 2.85 [95% CI, 1.24-6.56]). Cause-specific mortality risk (in absolute levels) generally remained higher for American Indian/Alaska Native arrestees across dispositions, except for cancer risk, which is statistically indistinguishable between the 2 races (eTable 3 in Supplement 1).

Finally, estimated variations for all-cause mortality were made by county type (Table 3). In urban counties, mortality risk is significantly elevated among White individuals sentenced to probation (IRR, 1.30 [95% CI, 1.18-1.42]), jail (IRR, 1.53 [95% CI, 1.36-1.73]), and prison (IRR, 1.29 [95% CI, 1.09-1.51]) relative to White individuals arrested only. Rural non–Indian Country counties were similar, with elevated White mortality risk for probation (IRR, 1.13 [95% CI, 1.02-1.26]) and jail sentences (IRR, 1.45 [95% CI, 1.13-1.86]). By contrast, White mortality risk was lower for those sentenced to prison (IRR, 0.93 [95% CI, 0.51-1.69]) in rural part–Indian Country.

**Table 3.** Regression-Adjusted Racial Mortality Disparities by Disposition and County Type^a^

Variable/interaction	All deaths by county type, incidence rate ratio (95% CI)		
	Urban	Rural	
		Non–Indian Country	Indian Country
Arrestees, No.	85 289	60 430	26 199
Person-years, No.	795 416	566 631	241 774
Disposition			
How mortality risk of White individuals differs by disposition relative to arrest only			
Fine	0.93 (0.75-1.14)	1.07 (0.83-1.36)	0.43 (0.29-0.62)^b^
Probation	1.30 (1.18-1.42)^b^	1.13 (1.02-1.26)^c^	0.82 (0.62-1.09)
Jail	1.53 (1.36-1.73)^b^	1.45 (1.13-1.86)^b^	1.06 (0.76-1.48)
Prison	1.29 (1.09-1.51)^b^	1.14 (0.91-1.43)	0.93 (0.51-1.69)
Racial disparities			
Baseline racial disparities for arrest-only reference disposition			
American Indian/Alaska Native individuals (arrest only)	2.70 (2.21-3.29)^b^	1.77 (1.29-2.44)^b^	1.72 (1.09-2.72)^c^
How baseline racial disparities are modified for each disposition			
Fine × American Indian/Alaska Native individuals	1.38 (1.17-1.62)^b^	0.68 (0.40-1.14)	2.72 (1.72-4.30)^b^
Probation × American Indian/Alaska Native individuals	0.60 (0.53-0.69)^b^	1.00 (0.65-1.54)	1.07 (0.78-1.46)
Jail × American Indian/Alaska Native individuals	0.76 (0.68-0.85)^b^	1.04 (0.72-1.50)	1.07 (0.72-1.59)
Prison × American Indian/Alaska Native individuals	0.45 (0.21-0.97)^c^	0.67 (0.32-1.39)	1.27 (0.57-2.81)

^a^ This table presents results from interacted Poisson regressions of mortality on race (reference group: White) and sentencing disposition (reference group: no disposition), controlling for sex, age group, offense type (drug/driving under the influence/violent/property), number of prior arrests, county type (urban/rural non–Indian Country/rural part–Indian Country), and arrest year. Standard errors were clustered at the county level.

^b^ *P* < .01.

^c^ *P* < .05.

In urban areas, mortality risk was 2.70 (95% CI, 2.21-3.29) times greater for American Indian/Alaska Native individuals relative to White individuals among those with arrest-only dispositions. The corresponding estimates are only 1.77 (95% CI, 1.29-2.44) times greater in rural non–Indian Country and 1.72 (95% CI, 1.09-2.72) times greater in rural part–Indian Country. In urban counties, disparities narrow among those sentenced to probation (IRR, 0.60 [95% CI, 0.53-0.69]), jail (IRR, 0.76 [95% CI, 0.68-0.85]), and prison (IRR, 0.45 [95% CI, 0.21-0.97]) relative to arrest only (in urban counties), but were exacerbated among those sentenced to fines (IRR, 1.38 [95% CI, 1.10-2.22]). There were no significant interactions in rural non–Indian Country counties and rural part–Indian Country counties, except for an increase in disparities among those sentenced to fines in rural part–Indian Country counties (IRR, 2.72 [95% CI, 1.72-4.30]), a result that is not significant in the sensitivity analyses. This suggests that the disparity reductions for probation, jail, and prison in Table 2 were driven by urban counties. The disparity calculations (eTable 4 in Supplement 1) suggest that although racial disparities are highest in urban counties for the reference disposition (ie, arrest only), the disparities are not statistically significant for prison and are similar to rural areas for probation and jail.

In the sensitivity analyses, the pattern of results for the main effects of race and disposition and the narrowing of disparities for probation and prison are generally similar to the main results (eTables 1 and 2 in Supplement 1).

## Discussion

To date, research has typically focused on mortality risk and racial disparities among current and recently released prisoners, typically not considering the role of place. The findings of our population-based study suggest that separately assessing the role of disposition, race, and place can mask important variations in mortality risk and racial disparities among justice system–involved populations.

Our study’s first finding highlights the importance of research and policy to address the needs of all justice system–involved persons given that even less severe dispositions had important consequences for mortality risk. Mortality risk among White individuals with probation and jail sentences was comparable to mortality risk for those with prison sentences.

The findings were even more nuanced for American Indian/Alaska Native arrestees. Our study confirmed that American Indian/Alaska Native individuals face greater mortality risk than their White counterparts overall, but it also provides new evidence that racial disparities are common to all disposition categories. That is, in the full sample, American Indian/Alaska Native individuals fare poorly relative to White individuals regardless of whether they are arrested and released, fined only, sentenced to probation in the community, jailed, or imprisoned.

However, the magnitudes of these disparities differed by disposition. Racial disparities narrowed substantially relative to arrest only, particularly for probation and prison. The disparity reduction for prison appears consistent with recent quasi-experimental work documenting “protective” effects of imprisonment on mortality, perhaps due to prisoners using health care services and other programs more the longer they served.^34,35^ Many prisoners also return to high-risk environments after incarceration, further pointing to the importance of place.^23,36,37^ The disparity reduction for probation does not appear to be the result of simply remaining in the community given that the reference group is the arrest-only disposition, who are also released in the community. We also observed no reduction for those sentenced to fines, who likewise remain in the community; fines may instead impose unintended consequences such as reducing financial resources available to pay for health care, food, and housing.^38,39^ Although the probation result could operate through community supervision, we caution that this mechanism is speculative as causal evidence on the impacts of probation—health or otherwise—is virtually absent from the literature. We further note the possibility that some probation sentences are revoked and result in a prison sentence. However, official statistics from South Dakota indicate that this happens infrequently, although questions about the integrity of these data remain.^40^

Our findings on cause-specific mortality further support this interpretation. Racial disparities in mortality due to external causes and cardiovascular/respiratory disease were significantly smaller for arrestees sentenced to probation and prison. These results again suggest that justice system–involved American Indian/Alaska Native individuals are often released back into the community and are at risk for elevated mortality due to external causes (eg, violence, accidents, and substance misuse) and cardiovascular/respiratory disease (eg, health care and lifestyle).^41,42,43,44,45,46^

Finally, the results of this study highlight the potentially critical role of place in shaping health and health disparities among this population. In urban areas, the role of dispositions appears more influential; although racial disparities for the arrest-only disposition were largest in urban counties, the narrowing of disparities across dispositions was also most evident in urban counties. This suggests that criminal justice policies may have an outsized impact on health outcomes for residents of urban areas.

### Limitations

The first limitation is that our results describe meaningful associations but not necessarily causation. Second, these data do not measure individuals’ socioeconomic characteristics, such as income, which may influence mortality. Similarly, this study used county type as a summary measure of place; thus, further work is needed to identify specific pathways, such as socioeconomic conditions, health infrastructure, and criminal justice systems. Third, these results are based only on South Dakota, which may limit their generalizability. Finally, race may be misclassified in criminal justice and death records, particularly for racially minoritized individuals.^47,48,49,50^

## Conclusions

The present population-based cohort study documented substantial racial disparities in mortality rates among arrestees, varying across disposition and place. We urge caution when considering the policy implications of these findings. American Indian/Alaska Native people have endured long-standing systemic racism as well as explicit and implicit bias that has impoverished many of these individuals and made them more likely to live in environments where mortality risk is elevated.^41,42,43^ Individuals in these communities often face limited access to health care and other disadvantageous social determinants that contribute to higher mortality risk.^44,45,46^ Further research is needed to understand the mechanisms through which dispositions and place shape these disparities.

## Notes

Supplement 1. **Appendix 1.** Linking Criminal Justice Records and Mortality Records in South Dakota **Appendix 2.** Classification of Cause of Death **Appendix 3.** Classification of County Type **eFigure 1.** Map of Indian Country **Appendix 4.** Robustness Checks Using Alternative Specification Assigning Disposition to Most Recent Arrest **eFigure 2.** Adjusted Mortality Rates by Race and Sentencing Disposition **eFigure 3.** Observed versus Expected Mortality Rates by Race and Disposition **eTable 1.** Regression-adjusted Racial Mortality Disparities by Disposition and Cause of Death **eTable 2.** Regression-adjusted Racial Mortality Disparities by Disposition and County Type **Appendix 5.** Walkthrough of Disparity Calculations **eTable 3.** Racial Disparities in Mortality by Disposition and Cause of Death. **eTable 4.** Racial Disparities in Mortality by Disposition and County

Supplement 2.
Data Sharing Statement
