# Supplementary material for: Mortality Disparities Among Arrestees by Race, Sentencing Disposition, and Place
Source: JAMA Health Forum. 2024 Jul 12;5(7):e241794. doi: 10.1001/jamahealthforum.2024.1794 (PMC11245725; doi:10.1001/jamahealthforum.2024.1794)
Supplement: Supplement 1. — Appendix 1. Linking Criminal Justice Records and Mortality Records in South Dakota Appendix 2. Classification of Cause of Death Appendix 3. Classification of County Type eFigure 1. Map of Indian Country Appendix 4. Robustness Checks Using Alternative Specification Assigning Disposition to Most Recent Arrest eFigure 2. Adjusted Mortality Rates by Race and Sentencing Disposition eFigure 3. Observed versus Expected Mortality Rates by Race and Disposition eTable 1. Regression-adjusted Racial Mortality Disparities by Disposition and Cause of Death eTable 2. Regression-adjusted Racial Mortality Disparities by Disposition and County Type Appendix 5. Walkthrough of Disparity Calculations eTable 3. Racial Disparities in Mortality by Disposition and Cause of Death. eTable 4. Racial Disparities in Mortality by Disposition and County [file jamahealthforum-e241794-s001.pdf]

Supplemental Online Content

Zuo G, Kilmer B, Nicosia N. Mortality disparities among arrestees by race, sentencing disposition, and place. *JAMA Health Forum*. 2024;5(7):e241794. doi:10.1001/jamahealthforum.2024.1794

eAppendix 1. Linking Criminal Justice Records and Mortality Records in South Dakota

eAppendix 2. Classification of Cause of Death

eAppendix 3. Classification of County Type

eFigure 1. Map of Indian Country

eAppendix 4. Robustness Checks Using Alternative Specification Assigning Disposition to Most Recent Arrest

eFigure 2. Adjusted Mortality Rates by Race and Sentencing Disposition

eFigure 3. Observed versus Expected Mortality Rates by Race and Disposition

eTable 1. Regression-adjusted Racial Mortality Disparities by Disposition and Cause of Death

eTable 2. Regression-adjusted Racial Mortality Disparities by Disposition and County Type

eAppendix 5. Walkthrough of Disparity Calculations

eTable 3. Racial Disparities in Mortality by Disposition and Cause of Death .

eTable 4. Racial Disparities in Mortality by Disposition and County

This supplemental material has been provided by the authors to give readers additional information about their work.

eAppendix 1. Linking Criminal Justice Records and Mortality Records in South Dakota

Criminal records data from the South Dakota Attorney General's Office contain information on all arrests involving suspected offenders ages 18 and older. The record tracks each arrest from the moment of arrest through disposition and, when applicable, sentencing.

Mortality data were provided by the South Dakota Department of Health. These include both in-state and out-of-state matches (the latter of were provided to the state through inter-state cooperation). We did not have direct access to these data ourselves due to data use agreements.

We were only able to receive detailed information (death ICD-9, date, etc.) on death records for perfect matches (either an SSN match, or a match on first/last name plus date of birth). For fuzzy matches, we only know they were in the mortality datasets, which means they must have died between 2000 and 2016. For the purposes of our analysis, we restricted deaths to perfect matches (for which a year of death was available) and we dropped the 0.03% of records with a fuzzy merge.

Below is a breakdown of the type of match between the arrest and mortality data when we examine this among the 624,958 observations in the arrest/county dataset. Roughly 4.9% of the arrests were perfectly matched with the mortality data.

dth_matchtype	Frequency	Percent	Cumulative Frequency	Cumulative Percent
1.perfect on first/last name, ssn, dob	23463	3.75	23463	3.75
2.perfect on ssn, dob	3922	0.63	27385	4.38
3.perfect on ssn	2028	0.32	29413	4.71
4.perfect on first/last name, dob	1065	0.17	30478	4.88
5.fuzzy merge, good	183	0.03	30661	4.91
6.fuzzy merge, bad	576419	92.23	607080	97.14
9.arrested after last death in records	17878	2.86	624958	100.00

A similar breakdown at the person level is below (note: 1 person is dropped because of missing county data):

dth_matchtype	Frequency	Percent	Cumulative Frequency	Cumulative Percent
1.perfect on first/last name, ssn, dob	4675	2.31	4675	2.31
2.perfect on ssn, dob	695	0.34	5370	2.65
3.perfect on ssn	312	0.15	5682	2.80
4.perfect on first/last name, dob	441	0.22	6123	3.02
5.fuzzy merge, good	73	0.04	6196	3.06
6.fuzzy merge, bad	193724	95.62	199920	98.68
9.arrested after last death in records	2673	1.32	202593	100.00

© 2024 Zuo G et al. *JAMA Health Forum.*

eAppendix 2. Classification of Cause of Death

Outcome Category	ICD-10 Codes	Count
External Causes	External causes of morbidity (V00-Y99)	1,603
Circulatory and Respiratory Disease (CRD)	Diseases of the circulatory system (I00-I99)	1,311
	Diseases of the respiratory system (J00-J99)	341
Cancers	Neoplasms (C00-D49)	984
Other Deaths	Diseases of the digestive system (K00-K95)	589
	Endocrine, nutritional and metabolic diseases (E00-E89)	215
	Mental, Behavioral and Neurodevelopmental disorders (F01-F99)	172
	Certain infectious and parasitic diseases (A00-B99)	150
	Diseases of the nervous system (G00-G99)	106
	Diseases of the genitourinary system (N00-N99)	49
	Symptoms, signs and abnormal clinical and laboratory findings, not elsewhere classified (R00-R99)	49
	Diseases of the musculoskeletal system and connective tissue (M00- M99)	23
	Diseases of the blood and blood-forming organs and certain disorders involving the immune mechanism (D50-D89)	9
	Diseases of the skin and subcutaneous tissue (L00-L99)	9
	Congenital malformations, deformations and chromosomal abnormalities (Q00-Q99)	5
	Pregnancy, childbirth and the puerperium (O00-O9A)	3
	Diseases of the eye and adnexa (H00-H59)	0
	Diseases of the ear and mastoid process (H60-H95)	0
	Certain conditions originating in the perinatal period (P00-P96)	0
	Injury, poisoning and certain other consequences of external causes (S00-T88)	0
	Factors influencing health status and contact with health services (Z00-Z99)	0
	Codes for special purposes (U00-U85)	0
	Total	5,618

© 2024 Zuo G et al. *JAMA Health Forum.*

eAppendix 3. Classification of County Type

eFigure 1. Map of Indian Country

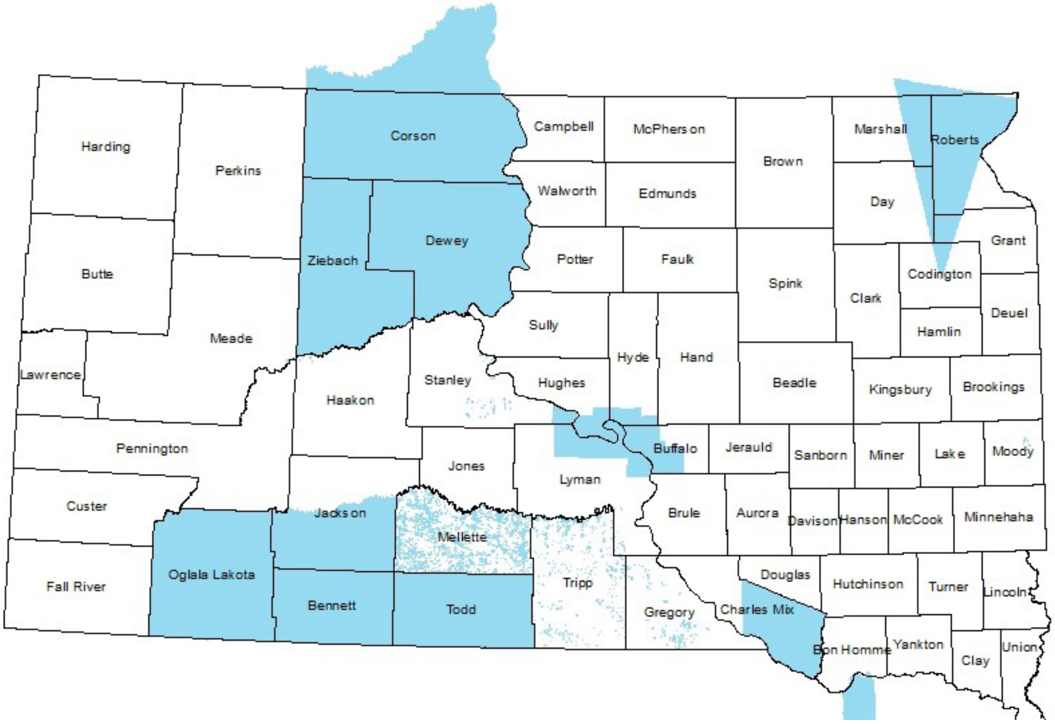

County type is a function of urbanicity and Indian Country (none, part, all). Our analysis also excluded arrests in the six counties comprised entirely of Indian Country, based on the federal definition of Indian Country, which includes federal Indian reservations and trust land allotments (18 USC § 1151, n.d.; 40 CFR § 171.3, n.d.) (see Appendix Figure C.1 for a map depicting Indian Country in South Dakota). This exclusion affected less than X% of arrests and aligns with our expectation given the “jurisdictional maze” that characterizes legal jurisdiction in Indian Country (Deloria & Lytle, 1983; Ulmer & Bradley, 2018, 2019). Jurisdiction for crimes occurring in South Dakota’s Indian Country lies with either tribal or federal courts when the defendant is American Indian (Leonhard, 2011). While crimes involving a non-American Indian defendant occurring in Indian Country may be prosecuted in federal or state court depending on the crime (Droske, 2007), our data demonstrate that the latter is rare.

© 2024 Zuo G et al. *JAMA Health Forum.*

eAppendix 4. Robustness Checks Using Alternative Specification Assigning Disposition to Most Recent Arrest

eFigure 2. Adjusted Mortality Rates by Race and Sentencing Disposition

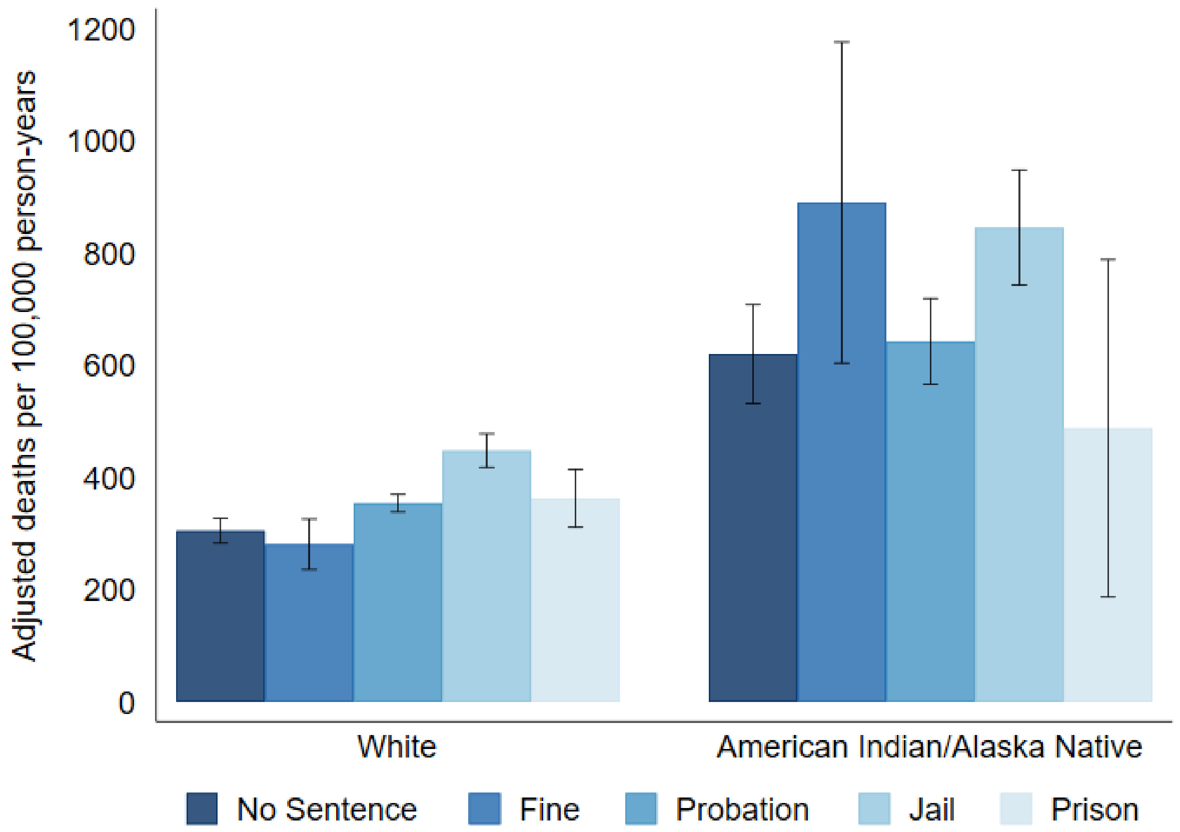

Note: This figure presents age- and sex-adjusted mortality rates (reference group: White) across five different sentencing dispositions between 2000 and 2016. Here, we use the disposition of an individual's most recent arrest instead of their first arrest.

© 2024 Zuo G et al. *JAMA Health Forum.*

eFigure 3. Observed versus Expected Mortality Rates by Race and Disposition

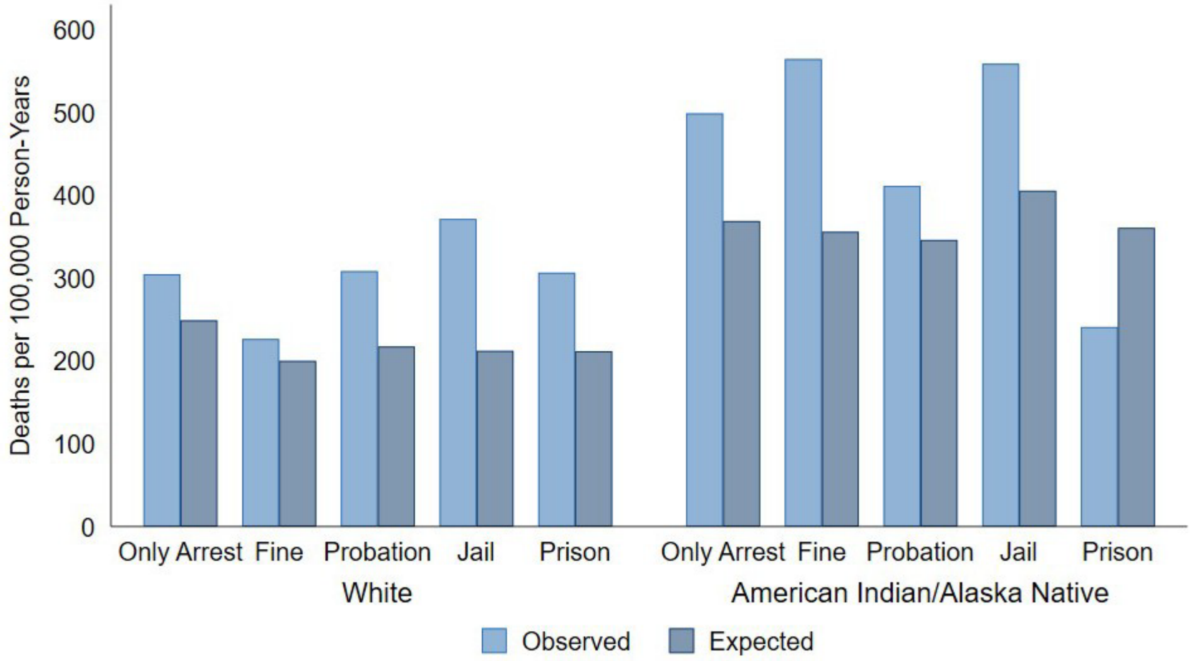

Note: This figure presents observed mortality rates by race and sentencing disposition for South Dakota's criminal justice population between 2000 and 2016, using the disposition of an individual's most recent arrest instead of their first arrest. Observed mortality rates are compared against expected mortality rates calculated based on the age, sex, and geographic composition for each subgroup and determining the expected number of deaths that would have occurred for an equivalent mix of age/gender/location in the broader South Dakota population. The analysis sample captures 314,987 unique arrests/disposition updates.

© 2024 Zuo G et al. *JAMA Health Forum.*

eTable 1. Regression-adjusted Racial Mortality Disparities by Disposition and Cause of Death

		Cause-specific Deaths			
	All Deaths	External Causes	Cancer	CV/Respir.	Other
Disposition					
How White mortality risk differs by disposition, relative to arrest-only					
Fine	0.941	0.785	0.798	1.249*	0.899
	[0.75,1.18]	[0.50,1.24]	[0.51,1.25]	[1.00,1.55]	[0.61,1.32]
Probation	1.188*	1.084	1.100	1.329**	1.209
	[1.03,1.37]	[0.89,1.31]	[0.93,1.30]	[1.08,1.64]	[0.99,1.47]
Jail	1.514**	1.432**	1.263*	1.751**	1.537**
	[1.32,1.74]	[1.19,1.72]	[1.02,1.57]	[1.36,2.26]	[1.25,1.89]
Prison	1.199*	1.091	1.055	1.526*	1.046
	[1.02,1.41]	[0.87,1.37]	[0.83,1.34]	[1.10,2.11]	[0.81,1.36]
Racial Disparities					
Baseline racial disparities for arrest-only reference disposition					
AI/AN	2.368**	2.576**	0.789	1.706**	4.256**
	[2.05,2.74]	[1.95,3.40]	[0.55,1.14]	[1.29,2.25]	[3.50,5.17]
How baseline racial disparities are modified for each disposition					
Fine x AI/AN	1.175	1.175	1.996	1.370	1.025
	[0.82,1.69]	[0.55,2.49]	[0.83,4.82]	[0.89,2.11]	[0.48,2.19]
Probation x AI/AN	0.779**	0.671**	1.410	0.873	0.744*
	[0.66,0.92]	[0.51,0.88]	[0.87,2.29]	[0.63,1.20]	[0.56,0.99]
Jail x AI/AN	0.811**	0.757*	1.116	0.930	0.757
	[0.71,0.93]	[0.59,0.97]	[0.77,1.61]	[0.65,1.33]	[0.56,1.02]
Prison x AI/AN	0.463**	0.397**	1.153	0.485*	0.457**
	[0.35,0.61]	[0.26,0.61]	[0.61,2.18]	[0.27,0.88]	[0.29,0.72]
# Arrestees	171,918	171,918	171,918	171,918	171,918
# Person-Years	1,603,821	1,603,821	1,603,821	1,603,821	1,603,821

Note: Sensitivity analysis sample captures 314,987 unique arrests/disposition updates, using the disposition of an individual's most recent arrest instead of their first arrest. This table presents results from interacted Poisson regressions of mortality on race (reference group: White) and sentencing disposition (reference group: no disposition), further controlling for sex, categorical age, offense type (drug/DUI/violent/property), number of prior arrests, county type, and arrest year. Standard errors were clustered at the county level. * p<0.05, ** p<0.01.
AI/AN: American Indian/Alaska Native

© 2024 Zuo G et al. *JAMA Health Forum.*

eTable 2. Regression-adjusted Racial Mortality Disparities by Disposition and County Type

	All Deaths, by County Type		
	Urban	Rural Non-Indian Country	Rural Indian Country
Disposition *(reference category: arrest-only disposition, conditional on White)*			
Fine	0.903	1.039	0.530*
	[0.65,1.25]	[0.79,1.36]	[0.30,0.95]
Probation	1.279*	1.125	0.971
	[1.03,1.59]	[0.95,1.33]	[0.76,1.24]
Jail	1.676**	1.393**	1.262
	[1.44,1.96]	[1.13,1.71]	[0.99,1.61]
Prison	1.340*	1.079	0.979
	[1.04,1.72]	[0.90,1.29]	[0.61,1.56]
Interaction *(how the racial disparity is modified for each disposition, relative to arrest only)*			
AI/AN (Arrest Only)	2.542**	1.786**	2.238**
	[2.15,3.00]	[1.29,2.47]	[1.44,3.48]
Fine x AI/AN	1.499**	0.994	1.512
	[1.13,2.00]	[0.38,2.63]	[0.87,2.62]
Probation x AI/AN	0.697**	1.062	0.875
	[0.63,0.77]	[0.59,1.90]	[0.64,1.20]
Jail x AI/AN	0.812**	1.013	0.791
	[0.71,0.93]	[0.72,1.43]	[0.58,1.08]
Prison x AI/AN	0.397**	0.491*	0.616
	[0.30,0.53]	[0.28,0.87]	[0.27,1.42]
# Arrestees	85,289	60,430	26,199
# Person-Years	795,416	566,631	241,774

Note: Sensitivity analysis sample captures 314,987 unique arrests/disposition updates, using the disposition of an individual's most recent arrest instead of their first arrest. This table presents results from an interacted Poisson regression of all-cause mortality on race (reference group: White) and sentencing outcome (reference group: no disposition), stratified by county type. The regression further controls for sex, categorical age, offense type(drug/DUI/violent/property), number of prior arrests, and arrest year. Standard errors were clustered at the county level. * p<0.05, ** p<0.01.
AI/AN: American Indian/Alaska Native

© 2024 Zuo G et al. *JAMA Health Forum.*

eAppendix 5. Walkthrough of Disparity Calculations

This Appendix briefly describes how one can estimate the size of the racial disparity between any two groups. We reference Table D1 below throughout this walkthrough which includes only estimates from Column 1 of Table 2.

- The “Prison” estimate in the first set of results (“Disposition”) shows the relative mortality risk for a White arrestee (the reference race) with a prison disposition relative to a White arrestee with an arrest-only disposition—in this case, 1.199x greater risk. The same logic applies for each successive estimate.
- The “Race” estimate in the second set of results shows the relative mortality risk of AI/AN arrestees with an arrest-only disposition (the reference disposition) relative to White arrestees with the same disposition—in this case, 2.368x greater risk.
- The “Prison x AI/AN” estimate in the “Interaction” section shows that for a given disposition among AI/AN (in this case, prison), the baseline AI/AN mortality gap of 2.368 is reduced by 0.463x.

These sets of estimates can be used to calculate the mortality disparity between any two combinations of race and disposition.

Suppose we want to calculate the difference in mortality risk between an AI/AN arrestee sentenced to probation versus a white arrestee sentenced to prison.

- We first calculate the mortality gap between the AI/AN+probation individual and a White+no- arrest individual.
  - For the AI/AN+probation individual, the “AI/AN” estimate is used (2.370), as is the “Probation” estimate (1.177). Since AI/AN=1 and Probation=1, it follows that the interaction AI/AN x Probation=1 (0.718). Since the Poisson regression output provides relative risk estimates, the three coefficients are multiplied together. The mortality gap between AI/AN+probation and White+no-arrest is therefore 2.368*1.188*0.718 = 2.020.
- We then calculate the mortality gap between a White+prison individual and a White+no- arrest individual. This is simply the first estimate in the “Disposition” section, or 1.194.
- To calculate the gap between AI/AN+probation and White+prison, we divide the two estimates, or 2.020/1.194 = 1.692.

The same logic can be used to calculate the disparity between any two combinations of race and disposition. We caution, however, that statistical significance does not carry through when making calculations of this sort. The “lincom” command in Stata can be used to calculate these specific standard errors.

© 2024 Zuo G et al. *JAMA Health Forum.*

eTable 3. Racial Disparities in Mortality by Disposition and Cause of Death .

		Cause-specific Deaths			
	All Deaths	External Causes	Cancer	CV/Respir.	Other
Relative Mortality Risk, AI/AN vs. White (Within Disposition)					
Arrest Only	2.37**	2.438**	0.686	1.872**	4.333**
Prison	1.494*	1.491	0.428	1.339	2.773**
Jail	1.916**	2.244**	0.966	1.522**	2.858**
Probation	1.700**	1.509**	1.071	1.256	3.199**
Fine	2.855**	2.786**	1.96	2.643**	3.906**
# Arrestees	171,918	171,918	171,918	171,918	171,918
# Person-Years	1,603,821	1,603,821	1,603,821	1,603,821	1,603,821

Note: This table presents results from interacted Poisson regressions of mortality on race (reference group: White) and sentencing disposition (reference group: no disposition), further controlling for sex, categorical age, offense type (drug/DUI/violent/property), number of prior arrests, county type, and arrest year. Standard errors were clustered at the county level. See Appendix B for a description of how causes of death were binned. * p<0.05, ** p<0.01.
AI/AN: American Indian/Alaska Native

© 2024 Zuo G et al. *JAMA Health Forum.*

eTable 4. Racial Disparities in Mortality by Disposition and County

	All Deaths, by County Type		
	Urban	Rural Non-Indian Country	Rural Indian Country
Relative Mortality Risk, AI/AN vs. White (Within Disposition)			
Arrest Only	2.701**	1.774**	1.722*
Prison	1.205	1.186	2.183*
Jail	2.052**	1.849**	1.842**
Probation	1.621**	1.779**	1.839**
Fine	3.724**	1.200	4.688**
# Arrestees	85,289	60,430	26,199
# Person-Years	795,416	566,631	241,774

Note: This table presents results from an interacted Poisson regression of all-cause mortality on race (reference group: White) and sentencing outcome (reference group: no disposition), estimated separately for three types of counties: urban, rural non-Indian Country, and rural Indian Country. The regression further controls for sex, categorical age, offense type (drug/DUI/violent/property), number of prior arrests, and arrest year. Standard errors were clustered at the county level.
AI/AN: American Indian/Alaska Native
* p<0.05, ** p<0.01.

© 2024 Zuo G et al. *JAMA Health Forum.*
