# Supplementary material for: Mortality Disparities Among Arrestees by Race, Sentencing Disposition, and Place
Source: JAMA Health Forum. 2024 Jul 12;5(7):e241794. doi: 10.1001/jamahealthforum.2024.1794 (PMC11245725; doi:10.1001/jamahealthforum.2024.1794)
Supplement: Supplement 2. — Data Sharing Statement [file jamahealthforum-e241794-s002.pdf]

Data Sharing Statement

Zuo. Mortality Disparities Among Arrestees by Race, Sentencing Disposition, and Place. *JAMA Health Forum*. Published July 12, 2024. doi:10.1001/jamahealthforum.2024.1794

Data
Data available: No

Additional Information
Explanation for why data not available: Our data are administrative criminal justice records linked to mortality records. We received these records directly from the state of South Dakota, and cannot make them publicly available.
